# On Polypi of the Heart as an Idiopathic Affection, and a Cause of Death

**Published:** 1831

**Authors:** 


### On Polypi of the Heart as an Idiopathic Affection, and a Cause of Death. By *W. Harty*, M.D. &c. &c.*

When we look around us and survey the intellectual and moral vigour of this most surprising age, and the strides which have been made within the last few years in almost every department of useful human knowledge, we cease to regret that the more recondite and abstract heights of science would seem to be less occupied in England than they were. The present times are distinguished for the general diffusion of moderate information, rather than for the brilliant achievements of individuals. We shall not stop to enquire if this be better or worse for mankind, as such discussion is wholly foreign to our purpose at present. What is true of the whole, must be true of the part, and thus it is that our own profession participates in the relations of the other classes of the civilized communities of the world. At present we have no imperator in science, but many are co-operating for the common good, and numbers are contributing to the accumulation of knowledge and experience. The cultivation of morbid anatomy has wrought an immense

* Dublin Medical Transactions, Vol. I. Part I.

change in the face of medicine and revolutionized its realms. We have derived already great benefit from investigation of the dead after study of the living, and, when the bulk of the profession have become yet better informed than they now are, when facts are more rightly observed and more accurately recorded, who knows where the chain of advantages may end, what useful discoveries may be made.

The diseases of the chest have received great attention in every age in which physic has been studied. Yet we might almost venture to affirm, that more has been done in the short period that has elapsed from the days of Corvisart to the present, than in fifty preceding centuries. This speaks volumes for morbid anatomy.

Polypi of the heart have long been a theme of contention with physicians and physiologists. It is not wonderful that in former times the most extravagant and idle notions were entertained upon the subject, nor is it to be marvelled at that modern researches should dissipate those unreal fancies. Yet we must not rush on too fast, we should not rashly exchange the extreme of credulity for the extreme of scepticism. If ancient physicians have seen in polypous concretions in the heart the cause of diseases which had no dependence on them, or connexion with them; many modern surgeons have precipitately asserted that their formation occurs only in the agonies of death. More researches, more facts are required to decide this point.

Dr. Harty, of Dublin, has endeavoured to add to the stock of information we possess, and relates some cases, calculated, in his opinion, to throw light on the investigation. We shall notice them with as much conciseness as possible.

*Case* 1. Miss R. æt. 14, of phthisical family, had been subject for some years to chorea, which had yielded to active purging. In December, 1814, she had a return of the disease, and, after a sudden alarm, it was aggravated with fever, palpitation, and dyspnœa. By free purgation and a bleeding to ℥xij. she was greatly benefited, and a relapse in February was successfully treated in the same manner. In March she was attacked with inflammatory sore throat, with night delirium, some dyspnœa and palpitation, and pain on pressure in the epigastric and right hypochondriac regions. She was bled and took calomel and James's powder with relief, though the action of the heart was still violent, and required digitalis with purgatives. In April, after exposure to cold, she was again attacked with fever, dyspnœa, palpitation, œdema of the face and a papulous eruption; the urine was scanty and high-coloured. A bleeding and blister procured some relief, but on the 4th she complained of cough, more dyspnœa, palpitation increased by lying on the left side. *Five leeches.* The bleeding from the leech-bites was very free, and she was relieved by them; she was ordered digitalis. On the 6th, the action of the heart strong, preferred lying on the right side. In the evening the pulse was 120, hard, and "communicated a peculiar thrilling, whizzing sensation to the finger, on touching any, the minutest artery in the body."* *Bled to a pint.* The blood was buffy, the pulse

* We suspect a little of the enthusiastic eloquence of the Emerald Isle in this passage.—*Rev.*

softer after the V. S. On the 7th the breathing was quick, the pulse 120. *Bled to* ℔j. *and again to* ℔j. *in the evening.* Lying on the left side excited incessant cough. On the 8th she had no pain, the cough was softer, the pupils dilated, and she had vomited. *A blister would not act.* In the evening the pulse was 106, hard, the skin hot and dry—*V. S. ad* ℥viij. *Another blister.* In the night she was thought to be dying, but next morning the respiration was not so panting; she now lay perfectly horizontal, and the palpitation was greatly increased by the erect posture. In the evening of the 10th she died.

On a hurried examination of the body, the lungs were found free from inflammation, and there was very little effusion into the pleural cavity. The liver was of large size. The pericardium exhibited some little appearance of inflammation, and contained about 6 ozs. of clear serum, without any lymph. The heart was hypertrophied and dilated, the vessels on its surface full of blood. A distinct polypus of a whitish colour, unconnected with any coagulum, nearly filled the right auricle and ventricle, its branches extending into the great vessels, adhering very slightly. A thick membranous substance, of the colour of the polypus, adhered with much firmness to the side of the ventricle opposite the septum, penetrating into its interstices, and binding down the valve. Both auricle and ventricle were of vivid red colour and "inflammatory aspect." The left ventricle was divided into two nearly equal cavities by an adventitious whitish membrane, firmly adhering to the internal apex, and to the sides of the ventricle, and terminating towards the aorta in a rounded organized polypus, which entered above an inch into the aorta. The two cavities into which the ventricle was thus divided communicated with each other very partially. The side of the membrane towards the left auricle was uneven, towards the aorta smooth. The auricle had the same inflammatory appearance as the right; its valves and those of the aorta were impeded by the polypus; three of the columnæ carneæ were much enlarged. The Doctor adds to the case a small row of queries.

"What was the nature and extent of the injury inflicted on the heart, in the first instance, by the terrors of the December storm? Was it then a general or partial affection of the organ, or was the nucleus of the disease then formed? Is there not under all the circumstances of the case reason to conclude, that the polypous membrane of the left ventricle had priority over that of the right, and that the great aggravation of the symptoms, together with the peculiar sensation imparted by the pulse, was caused by, or was concurrent with, the extension of the polypus into the aorta? Was inflammation or hypertrophy of the heart a cause or an effect of the polypous concretions?" 233.

*Case* 2. Master M. æt. 13, was attacked on the 14th Dec. 1818, with measles, then epidemic, and very fatal. He was twice bled to ℥viij. but on the morning of the 16th tracheitis suddenly came on, for which he was bled, leeched, blistered, and took calomel and James's powder. In the evening the face was livid and œdematous, the dyspnœa great, the pulse 140. *Warm bath—emetic—blister to sternum.* On the 17th the tracheitis was relieved, but there was pain in epigastrio, particularly on pressure. *Hirud.* viij. *epigast. Vesic. inter scap.* In the evening the respiration was more hurried, and there was palpitation for the first time. *V. S. ad* ℥vj.—*blister to throat—warm bath.* On the 18th, respiration was freer, but there was pain in the epigastrium. *V. S. ad* ℥vj.—*digitalis.* In the morning of the 19th the breathing was easier, he lay horizontal with ease, the eruption of measles had altogether disappeared; there was œdema of the face and slight emphysema of both arms. In the afternoon the skin was hot, the breathing more oppressed, and he complained of noise in the ears. *V. S. ad* ℥vj. The pulse now began to impart to the finger the peculiar wiry sensation noticed in the former case. *V. S. in the night, ad* ℥xj. Next day the thrilling was more distinct, "imparted by every artery" however small, and even felt through the thick muscles of the extremities; the arms and thorax were emphysematous. The medicines were now changed for purgation and the warm bath. On the 21st the chest, arms, and spine were emphysematous, and there were pain and soreness at the region of the heart, aggravated by inspiration. *Hirud.* vj.—*digitalis—emp. canth.* On the 22d he lay on the left side without uneasiness. Delirium now set in. On the 23d he lay horizontal, and was evidently sinking. In the morning of the 24th he died.

*Sectio Cadaveris.* "There was a considerable depression of the sternum, forming an arched hollow between the mammæ; a fact obvious to the eye during life. There was also unusual thickness of the sternum from the point of elevation upwards; there was slight adhesion of the left lung to the pleura in one or two points; also, a slight degree of adhesion near the junction of the two lobes; the external coat of the pericardium was evidently inflamed, though not to any great degree or extent, and there was about an ounce of serous fluid in the pericardium. The liver was enlarged in its dimensions, though by no means to the same extent as in the former case; it was otherwise apparently healthy, while the heart itself appeared larger than was compatible with his years, and the general structure of his frame. The left ventricle and auricle of the heart contained a large and singular polypus, unconnected with any coagulum, and adhering firmly in some parts, and more loosely in others. In the auricle (properly so called,) it adhered firmly throughout, maintaining a perfect union therewith by a number of lateral projections, and thence descending into the ventricle by a long and narrow neck, it formed a flat and firm adhesion to the side of the ventricle, throwing out at the same time a band, whereby it was connected to the polypous concretion which loosely occupied the apex and body of the ventricle, and extended thence into the aorta. The body of the auricular polypus branched largely into the pulmonary veins, and in its thickest portion contained a distinct, dense, and compact clot of blood, enveloped therein." 239.

Now it is on these cases that Dr. Harty builds his diagnosis of polypi in the heart. The symptom which he considers as peculiarly characteristic is the peculiar thrilling or whizzing sensation communicated to the finger by every artery in the body, as well as by the heart. When it occurs he concludes that the polypus has entered the great vessels issuing from the heart, and he ingeniously allows that polypus may exist in the heart without this diagnostic sign, *provided* it has not entered the valves. After some critical observations on the opinions of Senac, Bertin, and Laennec, Dr. Harty relates the case of Mr. Holder, referred to by John Bell, as "liker this disease than almost any other." It is given by Mr. Cheston, of Gloucester, in Simmons' London Medical Journal, for the year 1785. It will probably be new to our readers, and is worthy of perusal.

Mr. Holder was a surgeon-apothecary, in extensive and laborious practice, and had for four years before the fatal termination of his malady in 1775, experienced occasional pain and distress in the thorax on any great exertion—"of late however (i. e. a few months before death,) his pain extended farther into the right side and upwards towards his neck, where he was constantly sensible of a noise," similar to that of "a stream of water passing over obstructions, or forcing a passage through a narrow confined place." The gushing noise in the thorax became so strong and loud, that he often expressed his surprise at its not being heard—the pain at times was very great, and greatest when noise was least.—"That gush, he used to say, was his friend: while gush stood by him he should live." Pressure on the carotid artery relieved the noise, but it produced increase of pain. The pulse was hard and contracted, without the least intermission, and what was singular, the motion of the heart was not to be felt, though repeatedly searched for.—Having sustained a severe bilious attack, he experienced some remission of pain, and the apex of the heart was felt after it. As his strength declined, he found himself much more at ease by continuing almost constantly in an horizontal posture; he had good and bad nights with some regularity—on the latter, the motion of the heart was so languid, that he could not vary his position without the greatest caution; unless this was attended to, the circulation became confused, and, as it were, wholly carried on in one corner of the heart, which, at such times, beat with a "whizz." I omitted to mention that there was also "noise in the ear like dashing of water."

On dissection, the right auricle was found much enlarged and very thin, "with strong marks of inflammation," and the right ventricle nearly transparent. On opening them they were totally void of blood, and "a considerable quantity of air rushed out." At the upper part of the right ventricle from between the columnæ carneæ there arose a broad concretion, adhering firmly to the columnæ, about the thickness of half-a-crown, of a light yellow colour, very dense consistence, and occupying about two-thirds of the diameter of the cavity—then it rose into the auricle, and thence nearly four inches into the vena cava superior, one into the inferior, and also into the pulmonary artery; the branches not so firm as the main body. Another small concretion of darker colour, and more tender consistence, arose from the columnæ carneæ of the left ventricle, passing into the aorta about 1½ inch.

Such we may say broadly are the facts on which Dr. Harty grounds two separate propositions:—first, that polypi of the heart, exist as an idiopathic disease; and secondly, that the symptoms to which he and we have adverted are characteristic of its existence. Our own opinions on the subject may be of little consequence to others, but still, though perfectly open to conviction, we do entertain opinions. We believe that polypi may be formed in the heart for a longer or shorter time antecedent to death; but we do not consider that their existence as a distinct disease, *per se*, has yet been satisfactorily proved by Dr. Harty, nor, so far as we know, by any one else. Such is our conclusion from the premises, but the means of judging for themselves are before our readers and the profession in general.

We have said that polypi may exist in the heart, for a longer or a shorter time antecedent to death, and all who are practically conversant with morbid anatomy will allow that the period must be very various, its ascertainment extremely difficult. As a general law, we may assert that when patients are long in *dying*, polypi, presenting more or less of the appearances of maturity and age, will be found in the chambers of the heart. From simple black coagulum, we pass through all grades of their formation till we arrive at the brownish-red, lancellated substance, adhering firmly to the walls of the heart, almost apparently organized, even containing in its centre what has been, perhaps improperly, considered as pus. It cannot then be doubted or denied that polypi may be and are formed before the dissolution of the patient. Of the law which determines and regulates their formation we know little, but of the circumstances under which they are found we know more. They are witnessed in those who have been long in the agonies of death, who have been gradually sinking for hours or for days. A disease may be rapidly fatal and yet the act of dying may be long; a disease may be slowly mortal, and yet the final struggle may be brief. So far then, as we have seen, polypi have presented the characters of perfection and duration in proportion to the period during which the patient was sinking, and we think we have remarked that they are more mature when much depletion has been used. Supposing this last observation correct, it would be readily explicable on the ascertained principle, that blood-letting promotes the coagulation of the blood.

It may be asked, if we have any determinate symptoms produced by these formations. Now it is a singular and important fact, that of all who assert the existence of polypi as an independent disease, no two agree in the symptoms which they assign to it. Senac, Laennec, Bertin, differ; Mr. Cheston said a noise in the ear was the sign, Dr. Harty pronounces that it is a thrilling in the arteries. When none agree, common sense whispers that probably all are wrong. Dr. Harty endeavours to prove the inaccuracy of all former diagnostic marks; we will urge an objection or two to his; and then it remains for the next author to advance *his* certain sign, to be overthrown in a similar manner. A fact, to be admitted, requires two sorts of proofs, positive and negative. Let us test Dr. Harty's in this manner. In the most marked instance of polypous formation in the heart which we ever witnessed, we can positively assert that there was no thrilling whatever in the arteries. This is negative proof against Dr. Harty. Secondly, the peculiar thrilling in question is present in many cases of external aneurism, can be felt in all the tangible arteries of the body, and not unfrequently disappears altogether after the operation. It is also observed in hypertrophy of the heart and a roughened state of the coats of the aorta, and may exist for months and years. We have felt it in rheumatic pericarditis, diminishing or subsiding with the subsidence of the attack. This looks very like positive proof against Dr. Harty.

On the whole then, in the present state of our knowledge, it is safer not to consider polypi as a distinct disease, it is wiser not to pronounce upon their symptoms. When a patient is long in dying, that is, sinking, we may suspect that, they will be discovered, but farther than this our positive information scarcely extends. We do not wish to dogmatize. Dogmatism is the test of error, the foe of truth. But rational scepticism is better, in all cases of inductive reasoning, than easy confidence or hasty faith.

